# Chronic pain and accidental acute toxicity deaths in Canada, 2016–2017

**DOI:** 10.24095/hpcdp.44.7/8.02

**Published:** 2024-07

**Authors:** Jeyasakthi Venugopal, Amanda VanSteelandt, Lindsey Yessick, Keltie Hamilton, Jean-Franois Leroux

**Affiliations:** 1 Substance-Related Harms Division, Health Promotion and Chronic Disease Prevention Branch, Public Health Agency of Canada, Ottawa, Ontario, Canada; 2 Chronic Pain Policy Team, Controlled Substances and Cannabis Branch, Health Canada, Ottawa, Ontario, Canada

**Keywords:** chronic pain, drug overdose, opioid overdose, opioid crisis, controlled substances, substance use, substance-related disorders, acute toxicity

## Abstract

**Introduction:**

Multiple Canadian jurisdictions have reported a pattern of chronic pain among people who died from substance-related acute toxicity. This study examined the prevalence and characteristics of those with chronic pain using data from a national study of people who died of accidental acute toxicity.

**Methods:**

A cross-sectional analysis of accidental substance-related acute toxicity deaths that occurred in Canada between 1 January 2016 and 31 December 2017 was conducted. The prevalence of pain and pain-related conditions were summarized as counts and percentages of the overall sample. Subgroups of people with and without a documented history of chronic pain were compared across sociodemographic characteristics, health history, contextual factors and substances involved.

**Results:**

From the overall sample (n = 7902), 1056 (13%) people had a history of chronic pain while 6366 (81%) had no documented history. Those with chronic pain tended to be older (40 years and older), unemployed, retired and/or receiving disability supports around the time of death. History of mental health conditions, trauma and surgery or injury was significantly more prevalent among people with chronic pain. Of the substances that most frequently contributed to death, opioids typically prescribed for pain (hydromorphone and oxycodone) were detected in toxicology more often among those with chronic pain than those without.

**Conclusion:**

Findings underscore the cross-cutting role of multiple comorbidities and unmanaged pain, which could compound the risk of acute toxicity death. Continued prioritization of harm reduction and regular patient engagement to assess ongoing needs are among the various opportunities for intervention.

**Highlights**

- Between 2016 and 2017, at least one in ten of the people in Canada who died from an accidental acute toxicity had a documented history of chronic pain.
- People with chronic pain tended to be older and with no formal source of income.
- Mental health challenges, trauma and a previous surgery or injury were significantly more common among people with chronic pain than those without.
- Almost all individuals with chronic pain accessed health care services in the year before their death.

## Introduction

Substance-related acute toxicity deaths are an ongoing, widespread and complex public health emergency in Canada.^1^ Although this emergency is strongly tied to the use of increasingly toxic illegally manufactured drugs,^1^ historic high rates of prescription opioid use for pain management also contributed to this crisis.^2^ In 2017, Canada had the second-highest rate of daily opioid consumption in the world.^3^

A history of chronic pain was identified in 36% of all opioid-related deaths in Alberta in 2017.^4^ In British Columbia, approximately 45% of the people who died from illicit drug acute toxicity in 2016 and 2017 had contacted health services for assistance with pain-related issues in the year before their death.^5^

Chronic pain is a widespread health concern and a major contributor to disability in Canada.^6^ Certain populations are at greater risk of chronic pain: those with chronic conditions (e.g. diabetic neuropathy), older adults, postsurgical patients, people who have experienced an injury, and others.^7^ There is also a significant mental health burden; concurrent symptoms of depression and anxiety, as well as suicidal ideation, are common among individuals with chronic pain.^8^ As with substance-related harms, the prevalence and severity of chronic pain is often higher in populations affected by social inequities and discrimination.^2^,^9^ However, critical limitations in the measurement of pain underestimate the true burden of chronic pain at the population level.^7^

In line with evidence on the increased risk of chronic pain for people who have experienced an injury, recent reports have outlined a link between substance-related acute toxicity deaths and employment in industries with a high risk of injury. In British Columbia, 52% of those employed at the time of death were employed in trades or transport or as equipment operators.^10^ A similar pattern was reported in Alberta (53%)4 and Ontario (approximately 30% worked in construction).^11^,^12^ More recent data from Ontario (2018–2020) suggest that among people who died of opioid toxicity, people who worked in construction were more likely to be employed around the time of their death than those without a history of employment in construction (57.7% vs. 11.7%).^13^ Of note, a history of chronic pain was common among both those with (37.2%) and without a history of employment in construction (37.9%).^13^

Unmanaged pain may lead to people seeking relief from pain using nonprescribed substances, substance use disorders^9^ and an increased risk of overdose, especially among people diagnosed with opioid use disorder.^14^ The estimated prevalence of chronic pain among people who use substances ranges from 31% to 55%.^9^ A recent systematic review observed wide variability in the prevalence of substance use disorder or substance use–related challenges among patients with chronic non-cancer pain; prevalence of current substance use disorder ranged from 3% to 48%, while 16% to 74% had a lifetime history of any substance use disorder.^15^ Similar rates of substance use disorder have been observed among patients with cancer (2% to 35%).^16^

Managing chronic pain is a particular challenge for people who use substances because of stigma and discrimination. A study in Vancouver, BC, found that 66.5% of a sample of people experiencing moderate to extreme pain who use substances reported being denied prescription analgesics by clinicians.^17^ Of those who were denied prescription analgesics, many resorted to buying the requested pain medication (40.1%), a different pain medication (34.9%) or heroin (32.9%) on the street (participants were able to report multiple actions taken and may have taken one or all of these actions).^17^ Use of nonpharmaceutical substances and diverted prescription medication has been increasingly implicated in the ongoing emergency of substance-related acute toxicity deaths in Canada.^1^,^12^

Taken together, the evidence suggests a consistent link between substance-related acute toxicity deaths and chronic pain, as well as a disproportionate burden of these deaths among people employed in construction and trades. 

In this study, we estimated the minimum national prevalence of pain, and specifically chronic pain, among those who died from accidental substance-related acute toxicity in Canada between 2016 and 2017. We also examined differences between those with and those without a documented history of chronic pain by (1) sociodemographic characteristics, co-occurring health conditions and other known risk factors; (2)health-related encounters leading up to death, including history of prescription medication; (3) circumstances surrounding death and opportunities for intervention; and (4) toxicology findings.

## Methods

***Ethics statement***

This study was reviewed and approved by the Public Health Agency of Canada Research Ethics Board (REB 2018-027P), the University of Manitoba Health Research Ethics Board (HS22710) and the Newfoundland and Labrador Health Research Ethics Board (20200153).

***Data sources***

This present study is a descriptive, cross-sectional analysis of those who died from accidental substance-related acute toxicity between 1 January 2016 and 31 December 2017 in Canada. Data were obtained from a retrospective review of coroner and medical examiner files to examine the characteristics, circumstances of death and substances involved among those who died from acute toxicity. Cases were defined as those who died from acute intoxication as a direct result of administering exogenous substance(s), with one or more of the substances involved being a drug or alcohol. Detailed information on data collection and study eligibility is published elsewhere.^18^

Where available, residential postal codes were linked to Statistics Canada’s Postal Code Conversion File Plus to obtain area-based neighbourhood income quintile after tax (QAATIPPE).^19^

***Study population***

Between 1 January 2016 and 31 December 2017, 7902 people died of accidental acute toxicity across all the provinces and territories in Canada. The people who died were stratified by history of chronic pain as follows:

- With a history of chronic pain (N=1056): Any person whose medical records and/or witness statements (from family or friends) mention any of the following around the time of death or in the past: chronic back pain; other pain disorder or chronic pain; long-term (>90 days) treatment with opioid(s) for pain; fibromyalgia; or arthritis. Fibromyalgia and arthritis were included as they are common conditions catalogued under “chronic primary pain” and “chronic musculoskeletal pain,” respectively, in the International Classification of Diseases 11th revision (ICD-11).^20^
- Without a history of chronic pain (N= 6366): Any person without a documented history of chronic pain (according to medical records or witness statements) and no documented history of any of the following conditions (which are often associated with chronic pain): cancer; stroke; vascular diseases; irritable bowel syndrome; inflammatory bowel disease; osteoporosis; chronic autoimmune disorders; or neurological disorders. Conditions associated with chronic pain were identified based on descriptions in the ICD-11.^20^ These conditions are not included in the subgroup with a history of chronic pain as it is not possible to differentiate chronic pain from other possible primary symptoms or concerns associated with these conditions.

Of the 7902 people who died, 480 had no documented history of chronic pain but did have a history of a specific condition associated with chronic pain; they were excluded from the comparison groups. It is possible that more people experienced chronic pain and/or medical conditions associated with chronic pain, but their histories were not documented in the death investigation files. 

***Variables***

The variables included in this study describe interactions with health services, current or recently prescribed medications up to 6months preceding death, sociodemographic factors, known risk factors and co-occurring conditions for substance-related harms, circumstances of death and the substances involved (refer to Table 1 for descriptions). 

**Table 1:** Descriptions of variables included in the analysis of people who died of accidental acute toxicity, Canada, 2016–2017

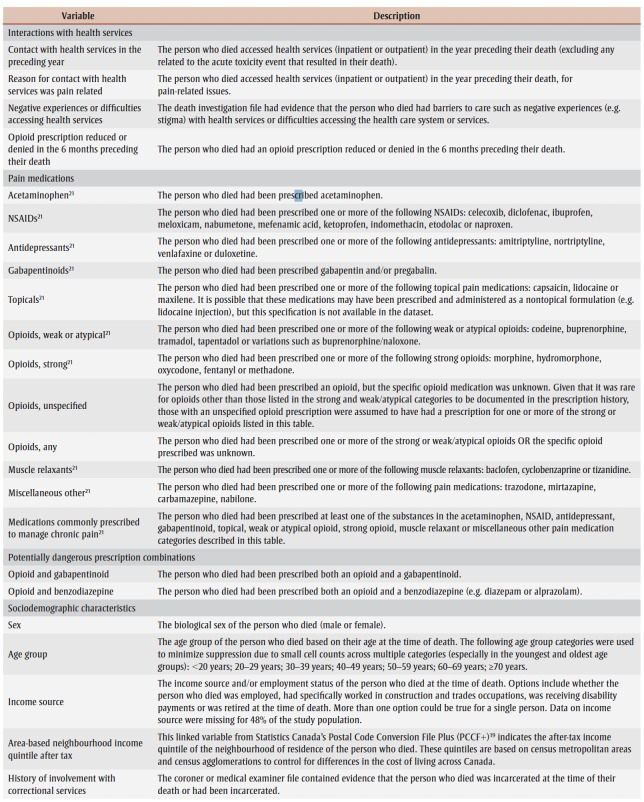 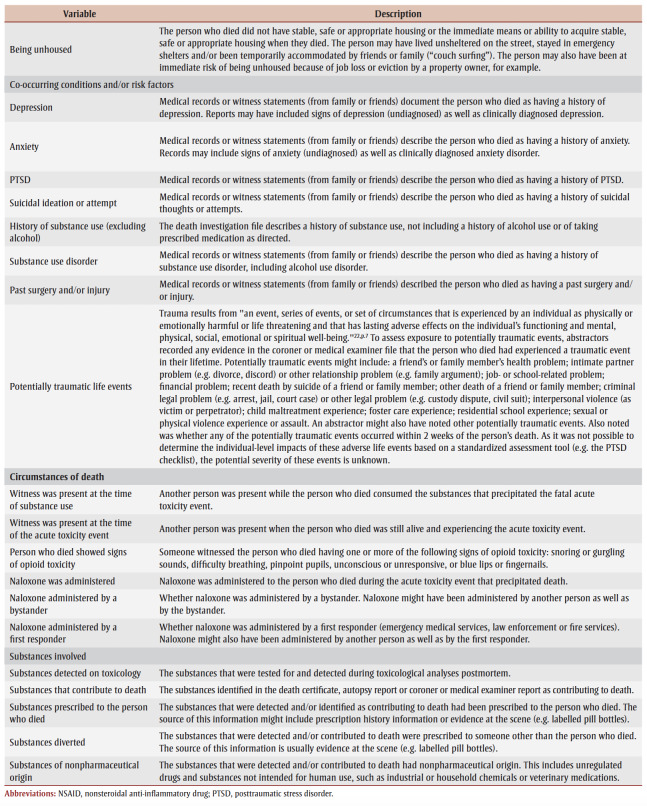

Specific medications prescribed for the management of chronic pain were identified based on the RxFiles *Pain Management & Opioids* mini-book.^21^ Variables indicating a prescription for opioids, chronic pain medications and any potentially dangerous combinations of medications (e.g. opioids and gabapentinoids or opioids and benzodiazepines)^21^ were included in this analysis because of their relevance to people with a history of chronic pain. Although available data on race, ethnicity and Indigeneity were extracted from death investigation files, these data are the focus of separate reports and are not included here.

Most of the variables were captured using “yes” and “no” values. Some were derived by coding “yes” and “no” for values captured in open text fields (e.g. pain medication names) or were categorical.

***Statistical analyses***

Frequencies and percentages for each variable were estimated for each subgroup. Statistically significant differences were identified through a Pearson chi-square test of independence. In accordance with privacy standards established for the original study,^18^ all counts shown in this paper were randomly rounded to base 3 and the percentages were based on these rounded counts. As subtotals and totals were rounded independently from their components, tables might not always sum to 100%. In addition, frequencies less than 10 were suppressed. 

All analyses and random rounding were performed using R statistical software version 4.2.1 (R Foundation for Statistical Computing, Vienna, AT).^23^,^24^ As this study is based on a chart review of death investigations, where information on a person’s entire life and medical history is not available, percentages represent the minimum proportions of people who had a given characteristic.

## Results

***Prevalence of pain among people who died of accidental acute toxicity***

Of the 7902 people who died of accidental substance-related acute toxicity in Canada between 1 January 2016 and 31 December 2017, at least 1056 (13%) had a documented history of chronic pain whereas 6366 (81%) had no documented history of chronic pain. The remaining 6% had no documented history of chronic pain, but did have a medical condition associated with chronic pain and might have belonged in either group (data not shown). Unspecified type of pain (17%), chronic pain or other pain disorder (8%) and back pain (6%) were the most frequently recorded types of pain (Table 2). For most people with a history of back pain, the pain was chronic (68%).

**Table 2:** History and types of pain among people who died of accidental substance-related acute toxicity, Canada, 2016–2017, N = 7902

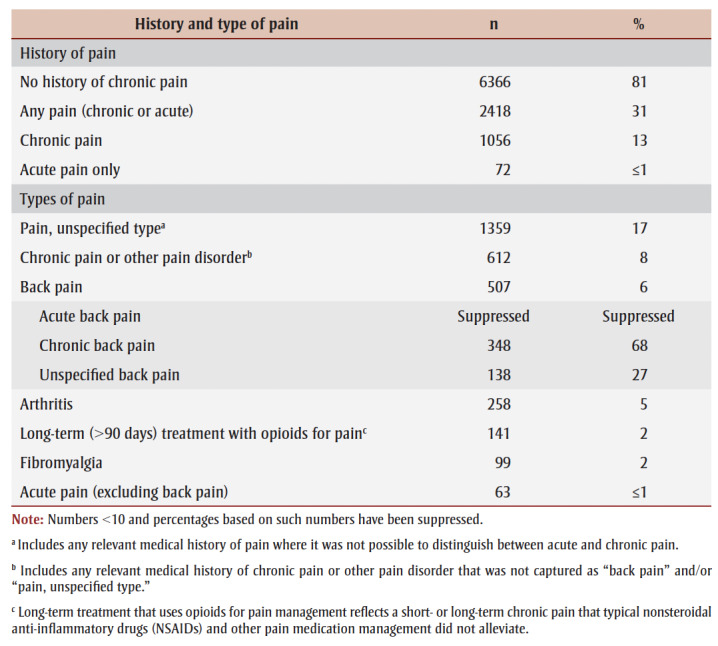

***Interactions with the health care systemand history of prescription medication***

There were significant differences between people with a history of chronic pain and those without for all interactions with the health care system and prescription medications examined (*p* < 0.05). People with a history of chronic pain had contact with the health care system in the year before their death more frequently (93%) than those without a history of chronic pain (65%). Moreover, the prevalence of contact with the health care system because of pain was almost 2 times higher among people with a history of chronic pain (30%) than among those without such a history (16%). Negative experiences or difficulty accessing the health care system (such as experiences of stigma) were also more prevalent among those with a history of chronic pain (4% vs. <1%). Some of these negative experiences may be related to difficulties accessing adequate pain management services, including pain medications (Table 3). 

**Table 3:** Interactions with the health care system and history of prescription medication among people who died of accidental substance-related acute toxicity, by history of chronic pain, Canada, 2016–2017

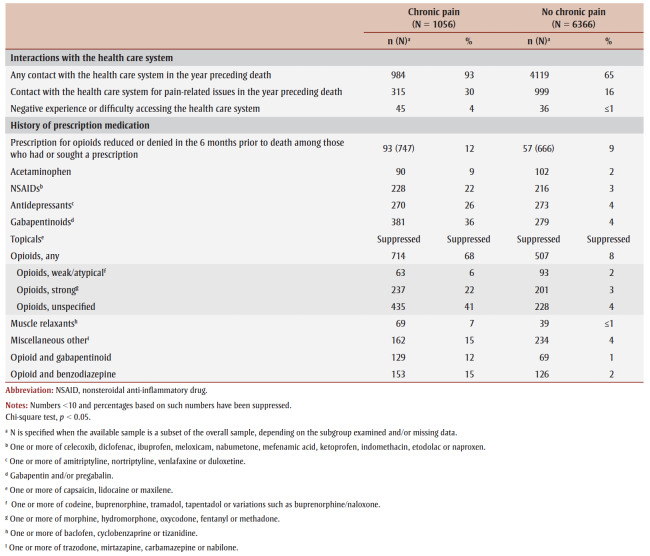

Among those who had or sought a prescription for opioid medication, those with a history of chronic pain had an opioid prescription reduced or denied in the 6 months prior to death more often (12%) than those without chronic pain (9%; *p*<0.05). Recent prescriptions for medications typically used for managing chronic pain and potentially dangerous prescription combinations of opioids with gabapentinoids or benzodiazepines were more prevalent among those with a history of chronic pain (12% and 15%, respectively, vs. 1% and 2%, respectively).

***Sociodemographic characteristics***

For most sociodemographic characteristics, co-occurring health conditions and other examined risk factors, differences were significant between people with a history of chronic pain and those without (*p*<0.05). Both those with and without a history of chronic pain were more often male; however, the proportion of males was higher among those without chronic pain (78%) than among those with chronic pain (57%) (Table 4).

**Table 4:** Sociodemographic characteristics, co-occurring health conditions, other known risk factors and circumstances surrounding death from accidental substance-related acute toxicity, by history of chronic pain, Canada, 2016–2017

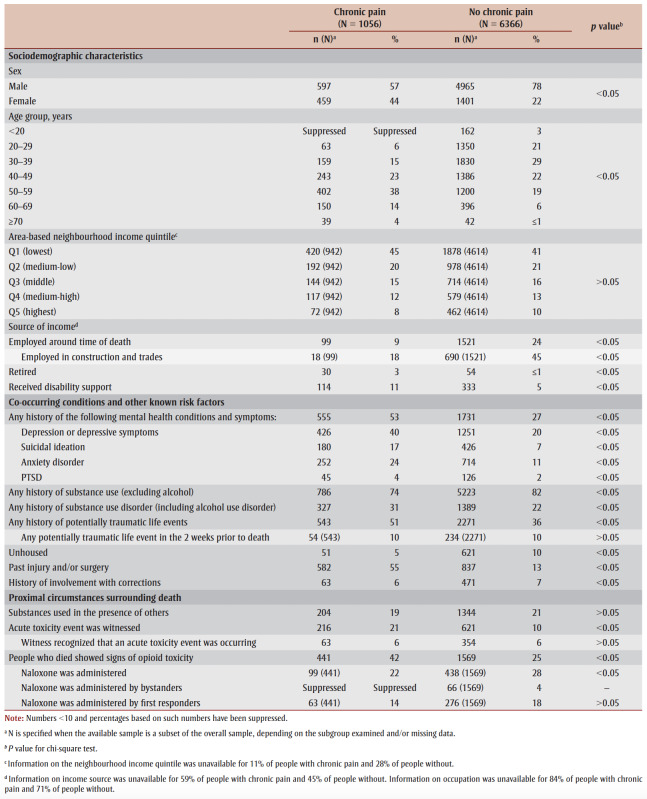

People with a history of chronic pain tended to be older; 56% were aged 50 years or older compared to 26% of those without chronic pain. Irrespective of their history of chronic pain, the majority of people who died from accidental substance-related acute toxicity lived in neighbourhoods in the lowest or medium-low-income quintiles. 

Among people with income source information, those with a history of chronic pain were less often employed (9%) than those without (24%). Among those who were employed, almost half of the people without a history of chronic pain worked in trades, construction or a related field. People with a history of chronic pain more commonly received disability support (11% vs. 5%) or were retired (3% vs. ≤1%). It is important to note that information on income source was unavailable for 59% of people with chronic pain and 45% of people without, and information on occupation was unavailable for 84% of people with chronic pain and 71% of people without.

***Co-occurring health conditions and other known risk factors***

More than half (53%) of all people with a history of chronic pain experienced a mental health condition compared to 27% of people without chronic pain (Table 4). Depression, anxiety, posttraumatic stress disorder and thoughts of suicide were all more prevalent among those with chronic pain. 

**Figure 1:**
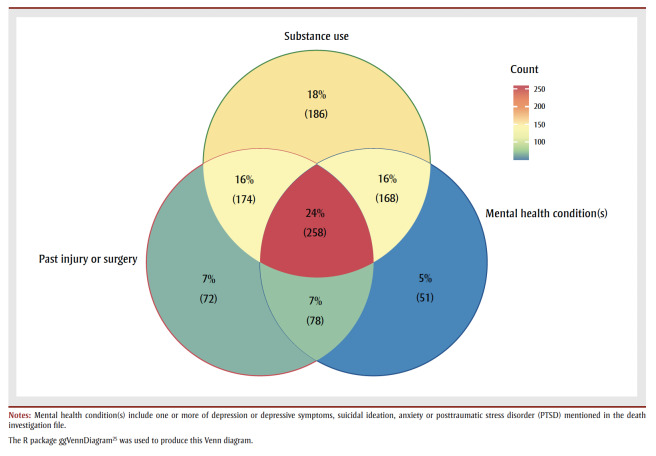
Venn diagram of the co-occurrence of a history of substance use (excluding alcohol), past injury and/or surgery and mental health conditions among people with a history of chronic pain who died of accidental acute toxicity, Canada, 2016–2017 (N = 1056)

While a history of substance use was more common among those without a history of chronic pain (82% vs. 74%), substance use disorder was more common among those with chronic pain (31% vs. 22%). More than half of all people with a history of chronic pain had experienced an injury or surgery (55%) compared to 13% of those without. About one in four people with a history of chronic pain had a combined history of substance use, a mental health condition and a past injury or surgery (Figure 1). In addition, those with a history of chronic pain more often had evidence of a potentially traumatic event in their lifetime (51%) than those without chronic pain (36%; Table 4). Experience of being unhoused were less common among people with a history of chronic pain.

***Circumstances surrounding death***

Similar proportions of people with and without a history of chronic pain used substances in the presence of others prior to the fatal acute toxicity event (Table 4). However, those with a history of chronic pain were more likely to have had a witness present at the time of death (21% vs. 10%). In addition, those with a history of chronic pain more often showed signs of opioid toxicity during the fatal event (42%) compared to those without (25%). 

Naloxone was more commonly administered for those without a history of chronic pain (18% vs. 14%).

***Substances involved***

Substances that contributed to more than 10% of deaths among people both with and without a history of chronic pain were fentanyl, cocaine, ethanol (alcohol), methamphetamine and morphine (Table5). Hydromorphone and oxycodone more often contributed to deaths of people with a history of chronic pain, while diacetylmorphine (heroin) and amphetamine more often contributed to deaths of those without. Opioids, which are frequently used to treat chronic pain, and other medications commonly used to treat chronic pain were among the substances that most frequently directly contributed to deaths of people in both groups. Potentially dangerous combinations of opioids with gabapentinoids or benzodiazepines were detected in the toxicology results of 20% and 43%, respectively, of people who had a history of chronic pain. These combinations were prescribed to more than half of people who died.

**Table 5:** Distribution of substances that contributed to most of the accidental acute toxicity deaths or that are associated with chronic pain, detection during toxicology testing, contribution to death, and substance origin, by history of chronic pain, Canada, 2016–2017

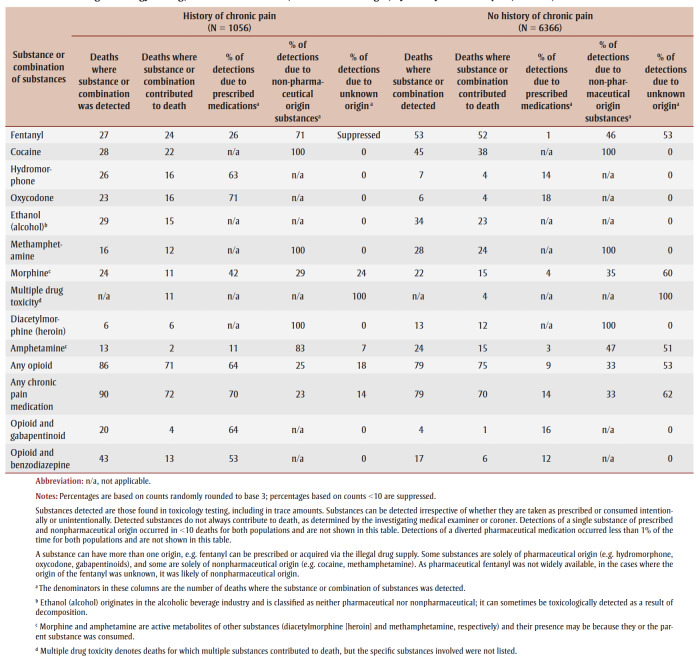

For all substances and combinations examined, people with a history of chronic pain were more commonly prescribed the substance that was detected in toxicology results than those without (Table5). For fentanyl and amphetamines, the lower percentage of detections due to substances of nonpharmaceutical origin among those without a history of chronic pain is likely due to higher percentages of substances having an unknown origin (not shown) when information about their medical history is also lacking. Detections of a diverted pharmaceutical medication occurred less than 1% of the time for both populations (data not shown).

## Discussion

The persisting high number of substance-related acute toxicity deaths in Canada continues to reflect the role of a toxic and unregulated drug supply1 within the broader context of factors influencing substance use and related harms. A pattern of injury and chronic pain among people who died from substance-related acute toxicity has been recorded in multiple jurisdictions in Canada.^4^,^5^,^10^-^12^ A history of chronic pain was documented in the coroner and medical examiner files for at least 13% of the people who died of accidental acute toxicity between 2016 and 2017.

Important differences in sociodemographic and other equity-relevant factors were noted between those with and those without a history of chronic pain. Most of those with a history of chronic pain were 40 years and older and resided in low- or medium-low-income neighbourhoods; compared with those without chronic pain, they were more often unemployed, receiving disability supports or retired at the time of death. These findings align with earlier work mapping the association between older age and lower socioeconomic status and increased prevalence of chronic pain and disability.^7^,^26^

Although there are reports linking substance-related acute toxicity deaths and employment in industries with a high risk of injury,^4^,^10^-^13^ we found employment in construction and trades to be more common among people with no history of chronic pain. This may be because of the overall lower prevalence of employment among people with chronic pain. However, this study was limited by the amount of missing information on employment history; as such, people who had been employed in trades (and incurred injuries leading to chronic pain or disability) may be undercaptured. Moreover, the seasonal and often time-limited nature of work in construction may serve as an accessible source of employment for people who use substances.^13^ The relationship between acute toxicity deaths and employment in construction and trades may also be underpinned by the mutual clustering of men in younger age groups.^13^ More research is needed to better characterize the association between employment in construction and trades and substance-related harms, taking into account the recency and duration of employment.

Closer examination of the potential cross-cutting role of multiple interrelated factors revealed considerable overlap between substance use, mental health conditions and past injury or surgery among those with chronic pain. Mental health conditions, history of trauma and past injury and/or surgery were significantly more prevalent among people with a history of chronic pain. These findings are unsurprising given the often-bidirectional association between pain, mental health and substance use–related issues. Rayner et al.^27^ reported that patients with depression were more likely to indicate heightened pain-related interference in daily functioning and more generalized pain. Traumatic events are also associated with an increased likelihood of functional somatic syndromes such as fibromyalgia.^28^ A history of trauma, such as maltreatment in childhood, and mental health conditions are also intricately linked and may result in greater pain severity and interference.^29^

Fentanyl was the leading contributor to death for both people with a history of chronic pain (24% of deaths) and those without (52% of deaths). When fentanyl contributed to death, people with a history of chronic pain were more often prescribed fentanyl (26%) than those without (1%). This suggests that fentanyl was frequently used for pain management among people with a history of chronic pain. However, the chart review dataset does not have data on the indications for the prescriptions that people were given. Since 2017, the last year of the study period, guidelines for the treatment of chronic non-cancer pain have recommended against opioid therapy,^21^,^30^,^31^ precipitating an expected decrease in the proportion of patients with chronic pain being prescribed fentanyl and other opioids. In contrast, the detection of fentanyl in drug seizure samples from law enforcement agencies has increased each year until 2021 in Canada, and it remains at high levels.^32^

The substances that most frequently directly contributed to death were more commonly prescribed to people with a history of chronic pain than those without, although nonpharmaceutical substances were also often detected in people with such a history. This does not mean that the prescriptions were necessarily inappropriate; the person who died may have taken more than their prescribed dose or supplemented or combined their medication with another pharmaceutical or nonpharmaceutical substance. 

In our study, we found that people with a history of chronic pain had opioid prescriptions reduced or denied in the 6months prior to death more often than those without such a history. Restricting access to pharmaceutical pain medications has been shown to steer people to illegal drug supplies, which are often more toxic and unpredictable.^17^ Of note, less than 1% of the substances that most frequently contributed to death were diverted prescription drugs. A harm reduction approach to prescribing for people with a history of chronic pain that emphasizes patient education about the substances they are prescribed and the potential risks of using other substances in combination with their prescriptions may reduce the risk of accidental death. People with a history of chronic pain had high contact rates (93%) with health services in the year preceding their deaths. About a third of the time, the contact was related to pain, providing opportunities for health care providers to review their patients’ prescriptions and talk about the use of pain medications and other approaches to pain management.

It is important that these opportunities for intervention not be missed through negative experiences such as prevailing stigma associated with chronic pain and substance use. In this study, people with a history of chronic pain had negative experiences when accessing health care services more often than those without such a history. These negative experiences further marginalize people who live with chronic pain and people who use opioids or other substances, possibly preventing them from receiving adequate services. Our findings are supported by those of a qualitative study examining the lived experiences of people who use substances; Dassieu et al.^33^ also describe the challenges in accessing interdisciplinary pain management services, the relative inaccessibility of nonpharmacological therapies for pain (such as physiotherapy) and the resultant potential for self-management with illegal drugs as a last resort.

***Strengths and limitations***

The chart review study is based on coroner and medical examiner files, which have different formats and investigation protocols across jurisdictions. The variables of interest in this analysis have different availabilities across jurisdictions; therefore, we are only able to present minimum proportions.

Capacity for toxicology testing also varies by jurisdiction and over time. The dataset does not include information about dosage and regimen duration, both of which contribute to the degree of risk for acute toxicity. However, the overseeing coroner or medical examiner would have assessed specifics on prescribed medications when determining the cause of death. Some medications have multiple on-label and off-label purposes; for example, buprenorphine, methadone and long-acting morphine can be prescribed for pain or opioid agonist therapy, and these opioids may have been misclassified as pain medications in the absence of information on indication(s) for use.

The ICD-11 diagnostic codes for chronic pain were published in 2018 and were not available to health care providers during the study period. In addition, some people living with chronic pain might not have sought a diagnosis or formal care, especially if they had previously faced barriers in accessing health care services (e.g. stigma). It is also possible that people with no known social contacts (such as family or friends) were missed if there was no one who could report on their experiences of chronic pain and/or barriers in accessing health care services.

Certain conditions associated with chronic pain (such as endometriosis) could not be separated from broader categories and were not excluded from the group with no history of chronic pain, resulting in some misclassification of people captured as having no history of chronic pain. In addition, a documented history of chronic pain may have influenced the collection of other medical and prescription histories. Given these limitations, differences observed between those with and those without a history of chronic pain are susceptible to bias. Furthermore, the cross-sectional nature of this study precludes causal inference.

Finally, it is important to note that the onset of the COVID-19 pandemic affected substance use patterns and harms.^34^ Accidental opioid toxicity deaths have almost doubled in Canada since March 2020 and remain higher than pre-pandemic trends, owing to various factors including the supply of increasingly unpredictable and toxic illegally manufactured drugs.^34^ While this present study examined a previously undescribed national population, future work should investigate the role of chronic pain in the sustained increase in substance-related acute toxicity deaths since the COVID-19 pandemic to guide policy planning and actions.

## Conclusion

Many cross-cutting and interacting factors likely influence the distinct burden of substance-related harms, including acute toxicity deaths, among people with chronic pain. Almost all the individuals with a documented history of chronic pain accessed health care services before their death, and almost a third of these interactions were for pain-related reasons. More than one in 10 people with chronic pain had an opioid prescription denied or reduced in the 6 months before their death. These findings signal unmanaged pain and the need for safe, adequate and accessible pain management solutions.

## Acknowledgements

We would like to acknowledge our collaborators at the offices of chief coroners and chief medical examiners across Canada for providing access to their death investigation files. We would also like to thank our co-investigators for their contributions in the development of the national chart review study on substance-related acute toxicity deaths: Brandi Abele, Matthew Bowes, Songul Bozat-Emre, Jessica Halverson, Dirk Huyer, Beth Jackson, Graham Jones, Fiona Kouyoumdjian, Jennifer Leason, Regan Murray, Erin Rees, Jenny Rotondo and Emily Schleihauf. 

This report is based on data and information compiled and provided by the offices of chief coroners and chief medical examiners across Canada. 

## Funding

This study was funded by the Public Health Agency of Canada.

## Conflicts of interest

The authors have no conflicts of interest.

## Authors’ contributions and statement

JV: Conceptualization, Formal analysis, Investigation, Writing – Original draft, Writing – Review & Editing.

AV: Conceptualization, Formal analysis, Investigation, Writing – Original Draft, Writing – Review & Editing.

LY: Conceptualization, Investigation, Writing– Review & Editing.

KH: Validation, Investigation, Writing – Review & Editing.

JFL: Conceptualization, Investigation, Writing – Review & Editing.

The content and views expressed in this article are those of the authors and do not necessarily reflect those of the Government of Canada or the data providers.
